# Possible association between the stage of HIV disease, antiretroviral treatment and the nutrient composition of breast milk in the Mangaung area, South Africa

**DOI:** 10.7448/IAS.17.4.19702

**Published:** 2014-11-02

**Authors:** Wilhelmina van den Heever, Geta de Wet, Moira Hattingh

**Affiliations:** 1 Health Sciences, Central University of Technology, Bloemfontein, Free State, South Africa; 2 Haematology, Pathcare Reference Laboratory, Bloemfontein, South Africa

## Abstract

**Introduction:**

In South Africa where replacement feeding may not be affordable, feasible or sustainable, HIV-infected women are recommended to exclusively breastfeed their infants during the first six months of life. The question arises whether HIV disease progression and its metabolic impact on the mother will affect the nutrient composition of breast milk. The aim was to determine the possible association between HIV disease progression, as measured by the immunological markers, and the nutrient composition of breast milk.

**Methods:**

The nutrient composition of breast milk of 100 HIV-infected and 50 non-infected volunteered (control group) lodging/day visiting mothers at Paediatric/Neonatal wards of National, Pelonomi and Universitas hospitals, Bloemfontein, were measured. The HIV-infected group was subdivided into HIV-naïve and HIV-ARV treatment group. Breast milk and blood samples were obtained. Macronutrients namely lactose, proteins, fat, total solids and the energy content of the breast milk and micronutrient namely calcium and phosphate were measured. Blood and immunological parameters comprised of CD4/CD8+T cell counts, viral loads and full blood counts.

**Results:**

Protein levels amongst the HIV-infected group showed a significant elevation (p<0.0001) compared to the control group. The calcium levels of the HIV-infected group were significantly lower (p=0.0081) than the control group. No statistically significant differences were recorded of the measured nutrients between mothers receiving treatment and the HIV-naïve group. All the HIV patients were anaemic with haemoglobin, haematocrit and RDW below the normal range. The Spearman Correlation Coefficient was used to determine if HIV disease progression have an influence on the nutrient composition. For the HIV-naïve group, a significant correlation was found between the viral load and percentage total solids in breast milk. A correlation between the CD4+T cell count, the percentage total solids and energy content of the breast milk was determined in the HIV-ARV treatment group. No strong positive correlation could be established between the immunological markers, HIV disease progression and the nutrient composition in the breast milk.

**Conclusions:**

HIV mothers can breastfeed their babies even at a more advanced stage of HIV disease progression, but emphasis has been placed on exclusive breastfeeding.

